# Function and fate of myofibroblasts after myocardial infarction

**DOI:** 10.1186/1755-1536-6-5

**Published:** 2013-03-01

**Authors:** Neil A Turner, Karen E Porter

**Affiliations:** 1 Division of Cardiovascular and Diabetes Research, and Multidisciplinary Cardiovascular Research Centre, School of Medicine, University of Leeds, Leeds LS2 9JT, UK

**Keywords:** Myofibroblasts, Heart, Myocardial infarction, Remodelling, Fibrosis

## Abstract

The importance of cardiac fibroblasts in the regulation of myocardial remodelling following myocardial infarction (MI) is becoming increasingly recognised. Studies over the last few decades have reinforced the concept that cardiac fibroblasts are much more than simple homeostatic regulators of extracellular matrix turnover, but are integrally involved in all aspects of the repair and remodelling of the heart that occurs following MI. The plasticity of fibroblasts is due in part to their ability to undergo differentiation into myofibroblasts. Myofibroblasts are specialised cells that possess a more contractile and synthetic phenotype than fibroblasts, enabling them to effectively repair and remodel the cardiac interstitium to manage the local devastation caused by MI. However, in addition to their key role in cardiac restoration and healing, persistence of myofibroblast activation can drive pathological fibrosis, resulting in arrhythmias, myocardial stiffness and progression to heart failure. The aim of this review is to give an appreciation of both the beneficial and detrimental roles of the myofibroblast in the remodelling heart, to describe some of the major regulatory mechanisms controlling myofibroblast differentiation including recent advances in the microRNA field, and to consider how this cell type could be exploited therapeutically.

## Review

## Introduction

At the cellular level, heart tissue constitutes cardiomyocytes, cardiac fibroblasts, vascular and neuronal cells, as well as inflammatory cells under certain pathological conditions. In the healthy heart, cardiac fibroblasts are the most prevalent cell type, accounting for up to 70% of cells, depending on the species in question [1,2]. Although cardiac fibroblasts have been much less well studied than cardiomyocytes, it is becoming increasingly apparent that the fibroblasts (and their differentiated phenotype, myofibroblasts) are integral to the development, normal function and repair of the heart, as well as contributing to adverse myocardial remodelling, fibrosis and heart failure progression [3,4]. Through physical and biochemical communication with myocytes and other cell types in the heart and the cardiac extracellular matrix (ECM), fibroblasts are well placed to sense and respond to stress or injury to the myocardium.

Fibroblasts are a heterogeneous population of cells, reflecting both their multiple developmental origins and their exposure to differential physical and chemical microenvironments. Fibroblasts derived from different anatomical sites have been proposed to effectively represent distinct differentiated cell types as they exhibit unique transcriptional signatures that probably reflect phenotypic differences [5]. Such diversity has made precise characterisation of fibroblasts challenging, and there remains no truly unique single marker that unequivocally identifies a cell as a fibroblast [6].

Although fibroblasts have the capacity to proliferate, migrate and regulate ECM turnover to maintain cardiac homeostasis, they are also able to undergo differentiation into a more contractile and synthetic myofibroblast phenotype to aid with cardiac repair following myocardial infarction (MI) [7-9]. Myofibroblasts are not normally found in the healthy myocardium, but are the most prevalent cell type in the infarct scar and are the main effectors of fibrogenesis [10]. Myofibroblasts are characterised by increased expression of particular contractile proteins (for example, α-smooth muscle actin, SMemb, vimentin), focal adhesion proteins (for example, paxillin, tensin, αVβ3 integrin), cell surface receptors (for example, transforming growth factor beta (TGF-β) type II receptor, angiotensin AT1 receptor, Frizzled-2), structural ECM proteins (collagen I, collagen III, fibronectin extra domain A splice variant (FN-ED-A)) and matricellular proteins (for example, periostin, osteopontin, tenascin C) [7-9]. Cardiac myofibroblasts are also highly proliferative, and those isolated from infarcted myocardium exhibit a higher rate of proliferation than cardiac fibroblasts from remote areas [11,12]. Although myofibroblasts are able to actively migrate to the infarcted region of the heart [13], a process regulated by Wnt/Frizzled signalling [14,15], they also appear to become less migratory as expression levels of contractile proteins increase [11,16]. Together these phenotypic changes confer increased tensile and ECM-secretory characteristics on the cells, enabling them to effectively facilitate the wound healing process.

### Beneficial and detrimental roles of myofibroblasts

**Figure 1:**
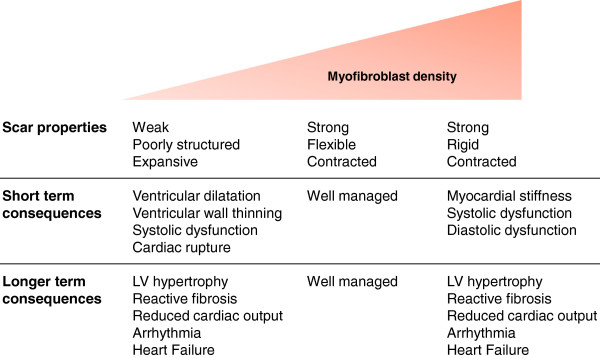
**Summary of the influence of myofibroblast density on post-myocardial infarction remodelling. **Low myofibroblast density in the infarct area results in a poorly structured, expansive and vulnerable scar that is prone to rupture or leads to systolic dysfunction and subsequent adverse myocardial remodelling. Although high myofibroblast density is important for a robust, contractile scar, excessive myofibroblast numbers (particularly in the remote myocardium away from the original infarct) drives fibrosis and myocardial stiffness, resulting in contractile dysfunction, arrhythmia and heart failure progression.

Appreciating the dual roles of cardiac myofibroblasts in the myocardial remodelling process is important, as they can be perceived to be both beneficial and detrimental depending on their prevalence and their temporal and spatial location. The infarct scar is not a simple acellular structure comprising structural ECM molecules; on the contrary, it contains myofibroblasts that maintain a viable, dynamic scar important for maintaining myocardial integrity against a background of continuous mechanical forces associated with the pumping of the heart [17]. Myofibroblasts are essential for rapid and robust (that is, strong and flexible) scar formation following MI. Interference with myofibroblast recruitment can result in infarct expansion, ventricular wall thinning, dilatation, systolic dysfunction and propensity to rupture [7] (Figure 1). Conversely, myofibroblast persistence can contribute to fibrosis and adverse myocardial remodelling, particularly if the myofibroblasts remain active in otherwise healthy areas of the heart away from the original site of injury (reactive fibrosis) [7]. Areas of increased ECM protein deposition can disturb the electrical conductance of the myocardium, thus increasing the likelihood of arrhythmias [18]. Moreover, direct coupling of cardiomyocytes to myofibroblasts, as opposed to fibroblasts, may also promote arrhythmias [19,20]. Fibrosis in the remote myocardium inevitably leads to increased myocardial stiffness, resulting in systolic and diastolic dysfunction, neurohormonal activation and, ultimately, heart failure [21,22] (Figure 1).

### Origin of myofibroblasts

The differential origin of myofibroblasts in the remodelling heart has become a hot topic in recent years [6,23]. Although once assumed to be solely derived from differentiation of resident fibroblasts, it is now apparent that cardiac myofibroblasts can also be derived from a multitude of alternative cellular precursors. These precursors include epithelial cells (through a process termed epithelial–mesenchymal transition), endothelial cells (through endothelial–mesenchymal transition; EndMT), mesenchymal stem cells, bone marrow-derived circulating progenitor cells (fibrocytes), smooth muscle cells and pericytes [6,23]. The recruitment of myofibroblasts from such diverse origins underlines their importance in the cardiac repair process, and probably represents optimised responses to different types of stress or injury. However, reports on the precise proportions of cells derived from different sources in different experimental models have varied considerably, so consensus has yet to be reached on the relative importance of myofibroblasts derived from resident cardiac fibroblasts versus extra-cardiac sources [6]. Another important aspect is whether these data are recapitulated in the human scenario. Nevertheless, a picture is now emerging that the source of myofibroblasts in the remodelling heart may depend heavily upon the nature of the initiating stimulus or injury. For example, whereas resident mesenchymal stem cells have been identified as important contributors to the myofibroblast population that drives post-MI scar formation, fibrocyte-derived myofibroblasts may be more important for interstitial fibrosis in the absence of MI [24]. Such knowledge opens up the exciting prospect that selective targeting of distinct myofibroblast populations could be used to protect essential repair mechanisms following MI, whilst reducing remote fibrosis and subsequent adverse myocardial remodelling.

### Factors stimulating myofibroblast differentiation

Phenotypic conversion of resident cardiac fibroblasts to myofibroblasts requires integration of both mechanical and biochemical stimuli. Fibroblasts are mechanosensitive and are therefore able to detect the loss of integrity of the ECM that occurs following MI. In response to increased mechanical stress and platelet-derived growth factor, fibroblasts adopt a partially differentiated phenotype known as the proto-myofibroblast [8]. Conversion of the proto-myofibroblast to the fully differentiated myofibroblast occurs in response to additional biochemical signals, particularly increased levels of active TGF-β and FN-ED-A [8], the levels of which are elevated in the damaged region of the heart post MI [25,26]. Such a phenotypic conversion is also promoted when cardiac fibroblasts are grown *in vitro* on rigid plastic surfaces; hence studies on cultured cardiac fibroblasts are generally indicative of myofibroblast behaviour [16,27]. TGF-β is normally present in the interstitium in a latent form, which can be rapidly activated by protease-mediated cleavage of the latency-associated peptide [28]. However, it has also been demonstrated that TGF-β activation can be stimulated directly by mechanical strain without the need for protease activity [29], and this mechanosensitive mechanism probably plays an important role in early myofibroblast conversion.

A number of additional stimuli that promote differentiation to the myofibroblast phenotype have been reported, including specific cytokines, growth factors and ECM molecules; several of which elicit their effects through up regulation of TGF-β activity and/or signalling [30]. There is also emerging evidence for an important role for the transient receptor potential family of ion channels in regulating cardiac myofibroblast differentiation. For example, the TRPM7 channel [31], the mechanosensitive TRPV4 channel [32] and the TRPC6 channel [33] have all recently been shown to be important for differentiation of cardiac fibroblasts *in vitro*. The latter study also employed an experimental MI model with TRPC6 knockout mice to show that myofibroblast differentiation was attenuated *in vivo* and this manifested in reduced infarct size, increased ventricular dilatation, reduced cardiac function and increased mortality due to ventricular wall rupture [33].

TGF-β-induced myofibroblast differentiation can be opposed by proinflammatory cytokines (for example, TNFα, IL-1) that may contribute to the temporal and spatial regulation of myofibroblast function in the transition from inflammatory to granulation and maturation phases of infarct healing [34]. Basic fibroblast growth factor can also inhibit TGF-β-induced myofibroblast differentiation, and was recently identified as an important paracrine factor that led to improved cardiac function following cell therapy in a rat MI model [35].

### Factors regulating myofibroblast persistence

Although myofibroblasts play key roles in scar formation, in most tissues (for example, skin) they usually undergo apoptotic cell death once the scar has matured and the healing process is resolved [36]. In the heart, however, whilst the density of scar myofibroblasts decreases rapidly in the weeks following MI [37-40], significant numbers can persist for many years [41]. A major driver of myofibroblast apoptosis in the heart and other tissues is thought to be a release from mechanical stress [42]. Repair of the damaged tissue with an organised cross-linked collagen-based ECM shields the myofibroblasts from mechanical stress, triggering the cells to proceed down an apoptotic pathway [42]. Additionally, cardiac myofibroblasts express the Fas receptor, and Fas activation is important in scar myofibroblast apoptosis after MI [43]. Strategies aimed at reducing myofibroblast apoptosis have reported favourable effects on infarct scar healing. For example, inhibition of Fas/Fas ligand interaction in mice 3 days after MI reduced apoptosis of myofibroblasts and macrophages, resulting in a thick, contractile and highly cellularised scar and alleviation of cardiac dysfunction, heart failure progression and death [43].

Recent *in vitro* evidence obtained using porcine aortic valve myofibroblasts suggests that fully differentiated myofibroblasts may also have the capacity to revert back to quiescent fibroblasts when substrate rigidity is reduced [44]. Furthermore, manipulation of TGF-β-induced signalling molecules (for example, c-Ski) may also promote reversal of the myofibroblast phenotype [45]. These studies highlight the potential plasticity of the myofibroblast phenotype that could make it amenable to therapeutic exploitation in the heart.

Importantly, while reducing apoptosis of myofibroblasts in the scar may deliver short-term beneficial effects, persistence of myofibroblasts in remote regions of the heart away from the scar area is detrimental. This is particularly relevant to nonischaemic cardiac remodelling such as left ventricular hypertrophy associated with pressure overload, in which myofibroblast persistence drives a profibrotic state leading to ventricular wall stiffening, neurohormonal activation, systolic and diastolic dysfunction and, eventually, heart failure [46,47].

### Epigenetics and microRNAs

**Figure 2:**
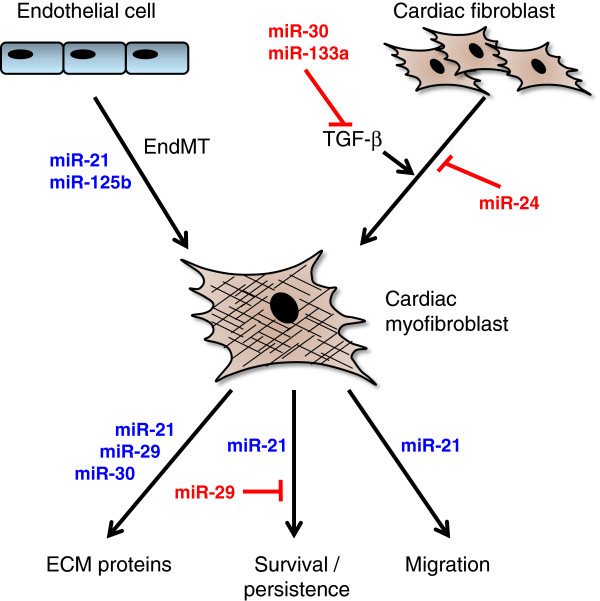
**Summary of potential roles of microRNAs in regulating cardiac myofibroblast phenotype and function. **microRNA (miR)-24, miR-30 and miR-133a inhibit transforming growth factor beta (TGF-β)-induced differentiation of resident cardiac myofibroblasts to myofibroblasts either directly or by reducing TGF-β levels. miR-21 and miR-125b stimulate conversion of endothelial cells to myofibroblasts via endothelial–mesenchymal transition (EndMT). Synthesis of extracellular matrix (ECM) proteins (for example, collagen) by myofibroblasts is upregulated by miR-21, miR-29 and miR-30, and miR-21 also stimulates cell migration and survival. In contrast, miR-29 inhibits cell survival. See main text for details.

Recent advances in a number of laboratories have revealed a role for epigenetics in influencing the differentiation process of myofibroblasts and resultant fibrogenesis (reviewed in [9]). These epigenetic influences include DNA methylation, post-translational histone modifications and regulatory noncoding RNAs, all of which can have profound effects on gene expression that control cell phenotype and function [48]. MicroRNAs (miRs) are the most widely investigated noncoding RNAs, acting as negative regulators of gene expression by inhibiting mRNA translation or promoting mRNA degradation [49]. There has been considerable interest in miR regulation of the myofibroblast phenotype in a variety of organs (reviewed in [50]). High-throughput screening approaches have enabled identification of miRs associated specifically with cardiac remodelling, and amongst those commonly reported are miR-133 (the most abundant in human heart), miR-1, miR-21, miR-29 and miR-208 (reviewed recently in [51]). Whilst early studies initially focused on the cardiomyocyte population, interesting roles for miRs specifically associated with cardiac fibroblasts and/or myofibroblasts are now emerging (Figure 2).

Differentiated myofibroblasts play central roles in fibrogenesis through their ability to synthesise increased quantities of ECM proteins. However, recent new data suggest that differential expression of miRs, and specifically elevated levels of miR-125b, can regulate the process of cardiac EndMT to a fibroblast-like phenotype in murine cells and promote profibrotic signalling [52]. Another study concluded that miR-21 contributes, at least in part, to EndMT in human umbilical vein endothelial cells [53]. Taken together these data support the idea that specific anti-miR targeting holds potential to ameliorate fibrosis by restricting the generation of myofibroblasts specifically derived through EndMT.

Differentiation of fibroblasts in the stressed myocardium confers ability to upregulate ECM turnover towards augmented fibrosis. A number of miRs have emerged as important regulators in this mechanism, and miR-21 and miR-29 have proven to be of particular interest. Whilst miR-21 expression is prominent in cardiac fibroblasts and substantially weaker in myocytes, increased miR-21 expression observed in failing murine hearts has been shown to be of fibroblast origin [54]. In that study, miR-21 targeting of Sprouty homologue, a negative regulator of the mitogen-activated protein kinase signalling pathway, led to increased fibroblast growth factor secretion, fibroblast survival and increased fibrosis [54]. In a different study, miR-21-mediated matrix metalloproteinase-2 expression in murine fibroblasts was reportedly via direct targeting of the phosphatase and tensin homologue, a negative regulator of the phosphatidylinositol 3′-kinase–AKT signalling pathway [55]. Whilst increased matrix metalloproteinase-2 secretion would imply increased degradation of ECM and fibroblast migratory activity, these processes were not directly investigated.

In the heart, miR-29 is mainly expressed in fibroblasts and has been shown to be downregulated in viable myocardium after experimental MI [56]. In the same study, the authors demonstrated that TGF-β stimulation of cultured cardiac fibroblasts downregulated miR-29 expression, suggesting that TGF-β drives the decrease in miR-29 in the remodelling heart. The first demonstration that miR-29 directly targets multiple ECM genes led to the proposal that miR-29 represses ECM gene expression in healthy myocardial fibroblasts and that miR-29 loss probably contributes to cardiac fibrosis by relieving this repression [56]. miR-29 has also been associated with apoptosis through modulating p53 activity [57], although this was demonstrated in a fibroblast cell line and would require validation in cardiac fibroblasts. Taken together these studies suggest that miR-29 therapeutics may be beneficial in regressing cardiac fibrosis.

An observed correlation between miR-24 expression and fibrosis in hypertrophic hearts was pursued in a recent study in which miR-24 was shown to be downregulated after MI and related to ECM remodelling [58]. Myocardial injection of miR-24 improved heart function and attenuated fibrosis and, notably, overexpression of miR-24 in cultured cardiac fibroblasts was also able to decrease differentiation to myofibroblasts through a reduction in TGF-β secretion and Smad phosphorylation [58].

In the healthy heart, it appears that expression of miR-133a and miR-30 are able to control ECM turnover by maintaining low secreted levels of the profibrotic cytokines TGF-β and connective tissue growth factor (CTGF/CCN2); a number of reports have demonstrated that miR-133a and miR-30 are downregulated in rodent and human heart failure [59,60]. miR-133a, whilst expressed only in myocytes and not fibroblasts, is believed to influence fibrosis through a paracrine mechanism that controls CTGF and TGF-β [60]. miR-30 is highly expressed in cardiac fibroblasts, however, and is believed to act in a manner similar to that of miR-29 – namely, by de-repression of profibrotic genes [60] – although whether fibrosis can be manipulated *in vivo* by miR-30 requires verification in functional studies.

### Therapeutic regulation

The challenge of post-MI therapeutic regulation at the level of the cardiac myofibroblast is to encourage optimum myofibroblast recruitment and retention in the scar (reparative fibrosis), whilst reducing myofibroblast activity in remote non-infarcted areas of the myocardium (reactive fibrosis). Several commonly prescribed drugs for MI patients that deliver beneficial effects on adverse cardiac remodelling appear to do so in part by directly influencing cardiac fibroblast and myofibroblast behaviour. Such drug classes include angiotensin-converting enzyme inhibitors, angiotensin receptor blockers, β-blockers, statins and thiazolidinediones (reviewed in [3,47,61]). However, regulation of cardiac (myo)fibroblast activity is not the primary target of these pharmacological agents, but instead appears to be an added pleiotropic benefit.

What about strategies designed specifically to target fibrogenesis? In light of our current knowledge of the mechanisms involved in post-MI repair and remodelling, some important considerations include: precisely controlling temporal administration of antifibrotic therapies to optimise scar formation, but ameliorate subsequent reactive fibrosis; targeting individual molecules that play differential roles in reparative and reactive fibrosis; and selective targeting of myofibroblast populations derived from different sources that play diverse roles in post-MI remodelling. Some such strategies are discussed briefly hereafter.

#### Transforming growth factor beta pathway

TGF-β is one of the most important inducers of the myofibroblast phenotype, as well as being an important profibrotic signalling molecule in its own right [28]. Inhibition of TGF-β activity before MI reduces the effectiveness of scar formation, leading to increased ventricular dilatation, decreased cardiac function and higher mortality rates, whereas TGF-β inhibition at later times after MI reduces adverse reactive fibrosis [62,63]. Because of the ubiquitous role of TGF-β in regulating tissue fibrosis, more selective strategies have attempted to target specific components of the TGF-β signalling pathway. For example, knockout of Smad3 increased myofibroblast density in the infarct scar in mice, whilst reducing interstitial myofibroblast numbers, decreasing dilatation and improving cardiac function compared with wild-type animals [64]. Other regulators of TGF-β signalling that have been proposed as therapeutic targets include the proto-oncogene c-Ski, the basic helix–loop–helix transcription factor Scleraxis and the proteasome E3 ligase Arkadia [65,66].

#### Matricellular proteins

The ability of TGF-β to induce fibroblast differentiation and fibrogenesis can also be regulated by matricellular proteins; a group of ECM-associated regulatory proteins that are expressed only under pathological conditions in the heart [42]. Ongoing studies suggest that some of these proteins may be viable therapeutic targets for regulating TGF-β activity and its downstream consequences, including myofibroblast accumulation and activation [42]. For example, tenascin C appears to be important for myofibroblast recruitment (differentiation and migration) to injured areas of the heart [67], but also contributes to adverse ventricular remodelling, fibrosis and heart failure after MI [68]. Periostin is important for infarct healing by promoting myofibroblast recruitment and collagen synthesis [69,70]. Periostin knockout mice subjected to experimental MI had increased rates of cardiac rupture, although those that survived had less fibrosis and improved cardiac function [69,70]. Thrombospondin-1 may help to limit the inflammatory phase of infarct healing and prevent the damaged area spreading to non-infarcted tissue [71], as well as being necessary for myofibroblast maturation and fibrogenesis in the pressure-overloaded heart [72]. CTGF (CCN2) is a multi-functional matricellular protein whose levels are elevated in both myocytes and myofibroblasts in the infarcted zone after experimental MI [73]. CTGF enhances TGF-β-induced fibroblast differentiation to myofibroblasts and appears to play an important role in neovascularisation [74]. CTGF probably plays a critical role in post-MI fibrosis, but such assumptions are based largely on observational data and therefore further studies with CTGF inhibition/knockdown are required to more precisely define its importance in regulating myofibroblast function in this context [74]. Osteonectin (SPARC) is another matricellular protein that is important for infarct healing, as mice deficient in SPARC exhibited adverse healing and deficient collagen maturation after MI, leading to increased cardiac rupture and dysfunction [75]. In a separate mouse study, SPARC deletion improved cardiac function 3 days after MI, but the absence of SPARC also resulted in impaired fibroblast activation and attenuated the increase in ECM production [76].

#### Wnt/Frizzled pathway

The Wnt/Frizzled signalling pathway has been shown to be an important modulator of the migration and differentiation of cardiac fibroblasts *in vitro*[14]. Moreover, in a mouse model of MI, administration of a specific peptide antagonist of Frizzled increased myofibroblast numbers and revascularisation in the infarct area, prevented infarct expansion, improved cardiac function and prevented heart failure mortality [15]. The potential therapeutic value of the Wnt/Frizzled signalling axis has been extensively reviewed recently [77].

#### Fibronectin extra domain A splice variant

FN-ED-A is an important contributor to the innate inflammatory response, as well as being a major driver of myofibroblast differentiation [8,78]. FN-ED-A is upregulated in the infarct area and remote myocardium following MI [25]. In an MI model using FN-ED-A knockout mice, infarct collagen levels (reparative fibrosis) were comparable with those of wild-type mice; however, reactive fibrosis in the remote non-infarcted area was reduced compared with wild-type animals, and conferred improvements in systolic and diastolic function and mortality [25]. Targeting FN-ED-A may therefore be an attractive therapy that is selective for reactive, rather than reparative fibrosis.

#### Myocardin-related transcription factor A

Myocardin-related transcription factor A (MRTF-A) appears to be a key inducer of gene programmes that mediate both cardiomyocyte hypertrophy [79] and fibroblast differentiation and fibrosis [80]. MRTF-A knockout mice exhibited a marked reduction in MI scar size with less myofibroblasts, but no detrimental effect on cardiac rupture or mortality [80]. Angiotensin II-induced reactive fibrosis was reduced in MRTF-A deficient mice compared with wild-type littermates [80]. MRTF-A may thus represent another potential therapeutic target for reducing adverse cardiac remodelling without compromising infarct scar healing.

#### Targeting different myofibroblast subsets

As discussed earlier, myofibroblasts in the remodelling heart are derived not only from resident cardiac fibroblasts, but also from endothelial cells (via EndMT), epithelial cells, mesenchymal stem cells, bone marrow-derived fibrocytes, smooth muscle cells and pericytes [6,23]. Therapeutic manipulation of the mechanisms involved in recruiting myofibroblasts from these different sources may therefore hold potential for modulating cardiac remodelling under different pathological conditions.

For example, monocyte chemotactic protein 1 (MCP-1/CCL2) is important for fibrocyte recruitment [81]. Cardiac overexpression of MCP-1 improves post-MI cardiac function and remodelling, at least in part by increasing myofibroblast accumulation [82]. Furthermore, MCP-1 deletion in a murine angiotensin II infusion model of interstitial fibrosis was demonstrated to reduce the number of CD34^+^/CD45^+^ (that is, fibrocyte-derived) myofibroblasts with resultant loss of interstitial fibrosis [83]. Rho kinase (ROCK-1) has also been identified as an important molecule regulating MCP-1-induced differentiation of CD34^+^/CD45^+^ fibrocytes into myofibroblasts in an ischaemic cardiomyopathy model [84]. Hearts from ROCK-1 null mice exhibited reduced numbers of fibrocytes and myofibroblasts, accompanied by reduced fibrosis and reduced cardiac dysfunction compared with wild-type animals [84]. One should note, however, that chemokines such as MCP-1 have far-reaching activities that are fundamental to the post-MI inflammatory process (for example, macrophage recruitment and activity) [85], and thus their targeting affects processes that extend beyond simple modulation of myofibroblast derivation from fibrocytes. Also, as with all animal studies, an element of caution should be exercised when considering knockout mouse results in relation to the situation in humans. For example, marked differences in MCP-1 expression levels post MI have been noted between mice and humans [86].

Nevertheless, as our knowledge on the origins of myofibroblasts in the heart increases, this will hopefully reveal novel therapeutic targets in addition to those described above. For example, it would be interesting to determine the effects of modulating miR-125b, as this has been shown to be important for regulating EndMT in the heart [52]. Strategies to target miRs will be discussed in more detail below.

#### MicroRNAs

The development and/or progression of many human pathologies is now widely accepted to be attributed to dysregulation of miRs, and understanding their functional relevance will advance exploitation of these molecules as therapeutic targets. Moreover, the tightly regulated cell type specificity of miR expression makes these molecules amenable to modulating function of individual cell types. Whilst current pharmacological therapies used in the treatment of adverse cardiac remodelling and failure are known to retard its progression, mortality rates remain high and there is a clear need for new therapies [87]. Whilst traditional therapies normally focus on a single target (for example, AT1R, β-AR) [3], by their very nature miRs regulate multiple genes, often within similar molecular pathways and signalling cascades. As such, they have potential to influence complex networks that are activated by a single stimulus (reviewed in [88]). For example, the miR-29 family is remarkably influential in regulating mRNA expression of a variety of collagens [56]. On the contrary, the breadth of miR-mediated effects also brings potential for disrupting cellular function through unwanted side effects [89].

Molecular tools for manipulating miR levels (through inhibition or mimicry) have been an area of rapid development and ongoing refinement [88]. As discussed above, several promising miR targets have been identified that appear to regulate myofibroblast differentiation and/or function (Figure 2). Preclinical studies manipulating miR-21 and miR-29 have shown beneficial effects on post-MI cardiac remodelling in rodents. Specifically, a miR-29 mimetic has proven successful in a murine model of cardiac fibrosis [56] and miR-21 inhibition increased survival after MI [55].

Progressive expansion of our knowledge concerning dysregulation of miRs in cardiac (myo)fibroblast phenotype and function will undoubtedly lead to strategies that optimise targeted delivery of miR therapeutics. The ability to deliver therapies directly to selected cell types is indeed a realistic option for future medicine.

## Conclusions

Cardiac myofibroblasts represent a unique, yet developmentally diverse, population of cells that play key roles in post-MI infarct healing, but also in adverse cardiac remodelling, fibrosis and progression to heart failure. Improved understanding of not only the origins of myofibroblasts in the post-MI heart, but also the capacity to assign specific roles and regulatory mechanisms to them, creates optimism for the future that this multifunctional cell type can be manipulated therapeutically to optimise infarct scar formation, whilst ameliorating reactive fibrosis.

## Abbreviations

CTGF: Connective tissue growth factor; ECM: Extracellular matrix; EndMT: Endothelial–mesenchymal transition; FN-ED-A: Fibronectin extra domain A splice variant; IL: Interleukin; MCP-1: Monocyte chemotactic protein 1; MI: Myocardial infarction; miR: microRNA; MRTF-A: Myocardin-related transcription factor-A; TNF: Tumour necrosis factor; TGF-β: Transforming growth factor beta

## Competing interests

The authors declare that they have no competing interests.

## Authors’ contributions

Both NAT and KEP contributed to the writing of the manuscript and approved its final submission.
